# Are the preoperative albumin levels and the albumin to fibrinogen ratio the risk factors for acute infection after primary total joint arthroplasty?

**DOI:** 10.3389/fsurg.2022.1043242

**Published:** 2023-01-06

**Authors:** Boyi Jiang, Hong Xu, Jinwei Xie, Duan Wang, Qiang Gan, Zongke Zhou

**Affiliations:** ^1^ Department of Orthopaedic Surgery, West China Hospital, Sichuan University, Chengdu, China; ^2^ Department of Orthopedics, Chongqing General Hospital, Chongqing, China

**Keywords:** total joint arthroplasty, albumin, albumin to fibrinogen ratio, acute postoperative infection, risk factor

## Abstract

**Background:**

Acute infection, such as periprosthetic joint infection and superficial surgical site infection, after primary total joint arthroplasty (TJA) is a serious complication, and its risk factors remain controversial. This study aimed to identify the risk factors for acute infection after primary TJA, especially the serological indicators that reflect preoperative nutritional statuses, such as albumin level and albumin to fibrinogen ratio (AFR).

**Methods:**

We retrospectively reviewed patients who underwent elective primary hip or knee arthroplasty at our institution from 2009 to 2021. Potential risk factors of acute infection and demographic information were extracted from an electronic health record. Patients who suffered acute infection, such as PJI or SSI, after TJA were considered the study group. Non-infected patients were matched 1:2 with the study group according to sex, age, the involved joint (hip or knee), and year of surgery (control group). The variables of potential risk factors for acute postoperative infection (demographic characteristics, preoperative comorbidities and drug use, operative variables, and laboratory values) were collected and evaluated by regression analysis. Restrictive cubic spline regression analysis was also used to examine the relationship between preoperative serum albumin levels and acute postoperative infection.

**Results:**

We matched 162 non-infected patients with 81 patients who suffered from acute postoperative infection. Among the patients who suffered from acute infection within 90 days after TJA, 18 were diagnosed with periprosthetic joint infection and 63 with surgical site infection. Low albumin levels were strongly associated with acute postoperative infection (95% confidence interval, 0.822–0.980; *P* = 0.015). This risk increased as preoperative albumin levels decreased, with a negative dose-response relationship (*P*_overall_ = 0.002; *P*_nonlinear_ = 0.089). However, there was no significant association between the AFR and acute infection after primary TJA (*P* = 0.100).

**Conclusion:**

There is currently insufficient evidence to confirm the relationship between preoperative AFR and acute infection after elective primary TJA, while a lower preoperative albumin level is an independent risk factor for acute infection with a negative dose-response relationship. This suggests that optimal nutritional management may be benefited before elective primary TJA.

## Introduction

Total joint arthroplasty (TJA) has become a standard operation for treating various end-stage knee and hip joint diseases (1, 2). As the population ages, primary total hip arthroplasty (THA) is projected to increase to 635,000 procedures and primary total knee arthroplasty (TKA) to 1.26 million procedures in the USA by 2030 (3). Additionally, complications, such as infection after TJA, are also expected to increase sequentially. Postoperative infection of joint surgery includes periprosthetic joint infection (PJI) and superficial surgical site infection (SSI). Among them, PJI is one of the most severe complications after primary TJA, and it is accompanied by higher hospitalization costs, longer courses of treatment, and higher morbidity and mortality rates (4). Despite the considerable progress in preoperative optimization, PJI still occurs in up to 2% of primary TJAs (5). Acute postoperative infection occurs within 90 days after primary TJA (6). This infection aggravates the patient's physical pain and results in a heavier psychological burden for the poor recovery of their joint function, which greatly reduces their quality of life and satisfaction. Acute postoperative infection may be related to the patient's nutritional status or immunity (7–9). Therefore, identifying the risk factors for acute infection after primary TJA and optimizing the risk factors to reduce acute postoperative infection are important.

Preoperative malnutrition is a risk factor for increased complications after primary TJA (10, 11). At present, several single serological indicators or combined indicators have been reported to be useful to evaluate the preoperative nutritional status (9, 12). Among them, a low serum albumin level (<35 g/L) is considered one of the simplest and most widespread markers of malnutrition (13). Additionally, the albumin to fibrinogen ratio (AFR) is a biomarker for predicting the nutritional and inflammatory status and prognosis in patients with various malignancies (14, 15). Recently, a low AFR was found to be associated with postoperative infection after revision knee and hip joint arthroplasty (16). However, few studies have investigated the relationship of preoperative albumin levels and the AFR with acute infection after primary TJA.

Therefore, this study aimed to determine whether preoperative albumin levels and the AFR are risk factors for acute infection after primary TJA. We also investigated whether there is a negative dose-response relationship between preoperative albumin levels, the AFR, and acute postoperative infection. Furthermore, we examined whether other indicators, such as the red blood cell width, are risk factors for acute infection after primary TJA.

## Materials and methods

### Data source

**Figure 1:**
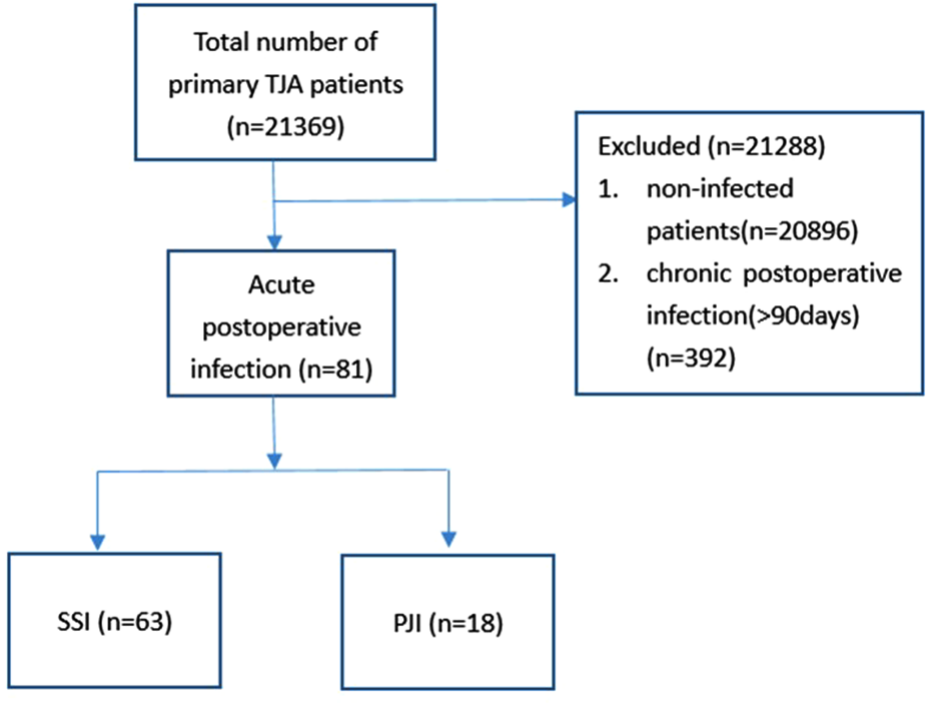
Flow chart showing the identification of the included infected group.

We performed a single-center, retrospective study using an electronic medical record system. We included patients who suffered from acute infection (<90 days) after primary unilateral THA or TKA in our hospital from 2008 to April 2021 (Figure 1). Our study design was approved and was considered exempt by the Ethics Committee on Biomedical Research, West China Hospital of Sichuan University (approval no. 2021-868). All methods were performed in accordance with the relevant guidelines, regulations and the Declaration of Helsinki. This study protocol was registered in the Chinese Clinical Trial Registry (Registration number: ChiCTR2100048385).

### Study design and data collection

In addition to the study group, we also included non-infected patients 90 days after primary THA or TKA who were matched 1:2 with the study group according to sex, age, the surgically treated joint (hip or knee), and the year of surgery as the control group. Age and sex were matched to reduce the effect of demographic characteristics on the study results. The year of surgery was also matched to reduce the effect of different perioperative management practices on infection rates in different years. We excluded patients with incomplete information.

All the surgical procedures were performed by five surgeons in our department, using the same approach (poster-lateral approach for THA and medial parapatellar approach for TKA). We performed standardized perioperative management for all patients. A dose of 20 mg/kg intravenous TXA (IV-TXA) was given before incision and another two doses of 10 mg/kg or 1 g IV-TXA 3 and 6 h later. We started using cefuroxime 1,500 mg 0.5–1 h before surgery and intravenously administered cefuroxime 1,500 mg again 8 and 16 h after surgery to prevent infection. Low-molecular-weight heparin daily, starting 6 h postoperatively and given until discharge, then oral rivaroxaban 5 or 10 mg daily for 10 days to prevent venous thrombosis. There was no tourniquet used during the operation. No drainage tube or early removal of drainage tube after surgery. All the patients performed ankle or hip flexion and extension exercises in bed immediately after surgery, then progressive ambulation exercises with full weight bearing on the first or second postoperative day with the assistance of a walker or crutches.

Each matching cohort (one patient with acute postoperative infection and two matched controls) was obtained on the basis of a stratification number in conditional regression modeling. We collected the following information: demographic characteristics, preoperative comorbidities, immunosuppressive drug use, operative variables, and laboratory values. Demographic characteristics comprised age, sex, body mass index, and orthopedic diagnoses, such as osteoarthritis and rheumatoid knee arthritis. The BMI of subjects were stratified into underweight (BMI < 18.5 kg/m^2^), normal weight (BMI 18.5–22.9 kg/m^2^) overweight (BMI 23.0–24.9 kg/m^2^) and obesity (BMI ≥ 25.0) groups (17). Preoperative comorbidities included hypertension, type 2 diabetes, chronic obstructive pulmonary disease, coronary heart disease, autoimmune disease such as ankylosing spondylitis and rheumatoid arthritis, and preoperative immunosuppressive drugs such as corticosteroids and cyclophosphamide. We recorded operative variables, such as the method of anesthesia and the American Society of Anesthesiologists class. We also collected preoperative laboratory test results that may be risk factors for acute postoperative infection. All patients completed the following laboratory tests 3 days before surgery. These laboratory tests were hemoglobin concentrations, the erythrocyte sedimentation rate, C-reactive protein concentrations, the red blood cell count, the standard deviation of red cell distribution width, the coefficient of variation of red cell distribution width, platelet count, white blood cell count, neutrophilic granulocyte count, lymphocyte count, monocyte count, and serum albumin, globulin, and fibrinogen concentrations. The AFR was calculated as follows: AFR = albumin (g/L)/fibrinogen (g/L). The albumin was detected using the colorimetric method, and the kit was produced by Roche Diagnostics GmbH, Germany. The fibrinogen was detected using the coagulation method, and the assay reagent was produced by Siemens Healthcare Diagnostics GmbH, Germany.

### Statistical analyses

Variables are shown as the mean ± standard deviation (SD) for continuous variables and the frequency (proportion) for categorical variables. A univariable analysis was performed to identify independent risk factors. A multivariable logistic regression analysis was carried out to evaluate the risk factors for acute postoperative infection in patients who underwent TJA. Odds ratios (ORs) for regression modeling and their 95% confidence intervals (CIs) were calculated. The level of significance level was set as *P* < 0.05. Data analyses were performed using IBM SPSS version 21 software (IBM Corp., Armonk, NY, USA). Furthermore, we used restrictive cubic spline regression analyses to detect relationships between preoperative albumin concentrations and acute postoperative infection using R version 3.6.1 (R Foundation for Statistical Computing).

## Results

### Patients’ demographics

Using the electronic medical record system in our hospital, we screened 21,369 primary TKA or THA, a total of 81 patients who suffered from acute postoperative infection were included, while 162 non-infected patients were matched. The acute postoperative infection rate was 0.38%. In the study group, 18 patients suffered from periprosthetic joint infections, and 63 suffered from SSI. Among them, 46 patients suffered from acute infection after TKA, and 35 patients suffered from acute infection after THA. Our population was predominantly women (69.14% vs. 30.86% men). The mean age at the time of surgery was (60.20 ± 11.02) years.

### Univariable analysis of risk factors for acute postoperative infection

We first investigated the potential risk factors for acute infection after primary TJA using a univariable analysis. This analysis showed that serum albumin levels (*P* = 0.003), C-reactive protein concentrations (*P* = 0.012), and autoimmune diseases (*P* = 0.047) were associated with acute postoperative infection. There was no significant association between the AFR and acute infection after primary TJA (*P* = 0.100) (Table 1).

**Table 1:** Baseline characteristics of the patients and univariable analysis of risk factors for acute postoperative infection after TKA or THA.

Perioperative factors	Acute postoperative infection (*n* = 81)	Non-infection (*n* = 162)	*P* value
BMI (kg/m^2^)			
Underweight	4 (4.94%)	7 (4.32%)	1.00 (reference)
Normal	26 (32.10%)	53 (32.72%)	1.00 (reference)
Overweight	18 (22.22%)	33 (20.37%)	1.00 (reference)
Obesity	33 (40.74%)	69 (42.59%)	0.980
ASA class			
I and II	69 (14.81%)	150 (92.59%)	1.00 (reference)
III and IV	12 (85.19%)	12 (7.41%)	0.068
Hypertension			
No	53 (65.43%)	111 (68.52%)	1.00 (reference)
Yes	28 (34.57%)	51 (31.48%)	0.628
Type 2 diabetes			
No	72 (88.89%)	143 (88.27%)	1.00 (reference)
Yes	9 (11.11%)	19 (11.73%)	0.887
Smoking			
No	71 (87.65%)	142 (87.65%)	1.00 (reference)
Yes	10 (12.35%)	20 (12.35%)	1.00
Alcohol abuse			
No	70 (86.42%)	139 (85.80%)	1.00 (reference)
Yes	11 (13.58%)	23 (14.20%)	0.896
Autoimmune disease			
No	67 (82.72%)	148 (91.36%)	1.00 (reference)
Yes	14 (17.28%)	14 (8.64%)	0.047*
Preoperative laboratories			
ESR (mm/h)	32.67 ± 24.29	27.58 ± 18.95	0.075
Hb (g/L)	129.31 ± 21.51	136.33 ± 36.79	0.114
RBC (10^12^/L)	4.36 ± 0.56	4.47 ± 0.58	0.161
RDW-SD (fl)	46.99 ± 4.78	45.85 ± 4.84	0.083
RDW-CV (fl)	13.98 ± 1.75	13.94 ± 1.16	0.860
PLT (10^9^/L)	186.69 ± 80.43	187.14 ± 68.14	0.964
WBC (10^9^/L)	6.15 ± 1.74	5.81 ± 1.72	0.145
NEUT# (10^9^/L)	3.99 ± 1.54	3.63 ± 1.32	0.060
LYMPH (10^9^/L)	1.62 ± 0.60	1.60 ± 0.59	0.736
MONO (10^9^/L)	0.36 ± 0.13	0.34 ± 0.13	0.122
ALB (g/L)	41.67 ± 4.42	43.41 ± 3.63	0.003*
Globulin (g/L)	27.75 ± 5.32	28.19 ± 16.25	0.813
Fibrinogen (g/L)	3.20 ± 0.91	3.06 ± 0.72	0.213
AFR	14.08 ± 4.27	14.99 ± 3.87	0.100
CRP (mg/L)	3.79 ± 15.71	2.96 ± 6.15	0.012*

ASA, American Society of Anesthesiologists; BMI, body mass index; ESR, erythrocyte sedimentation rate; Hb, hemoglobin; RBC, red blood cell count; RDW-SD, red cell volume distribution width- standard deviation; RDW-CV, red cell volume distribution width-coefficient of variation; PLT, platelet count; WBC, white blood cell count; NEUT, neutrophilic granulocyte count; LYMPH, lymphocytes count; MONO, monocyte count; ALB, albumin; AFR, albumin-fibrinogen ratio; CRP, C-reactive protein.

*P < 0.05.

### Multivariable analysis of risk factors for acute postoperative infection

We further examined the risk factors for acute infection using multivariable analysis by including the American Society of Anesthesiologists class, autoimmune disease, erythrocyte sedimentation rate, standard deviation of red cell distribution width, neutrophilic granulocyte count, albumin levels, AFR, and C-reactive protein concentrations, which had a *P* value <0.1 in the univariable analysis. We also accounted for age and sex as confounding variables in our multivariate logistic regression model. The Hosmer and Lemeshow Test showed that *P *= 0.929. It is considered that the information in the multivariable analysis has been fully extracted, and our model has a high goodness of fit. We found that only albumin levels were associated with acute postoperative infection (OR 0.897; 95% CI 0.822–0.980; *P* = 0.015). No associations were found between the AFR and other indicators and acute infection after primary TJA in multivariate analysis (OR 1.054; 95% CI 0.958–1.159; *P* = 0.283) (Table 2).

**Table 2:** Multivariable analysis of independent risk factors for acute postoperative infection after TKA or THA.

Perioperative factors	*P* value	OR	95% CI
ASA class			
I and II	1.00 (reference)	–	–
III and IV	0.300	0.587	(0.214–1.609)
Autoimmune disease			
No	1.00 (reference)	–	–
Yes	0.990	0.993	(0.369–2.674)
ESR	0.570	1.005	(0.988–1.021)
RDW-SD	0.692	1.014	(0.948–1.084)
NEUT	0.305	1.122	(0.901–1.398)
ALB	0.015	0.897	(0.822–0.980)
AFR	0.283	1.054	(0.958–1.159)
CRP	0.199	1.028	(0.985–1.073)
Age	0.235	0.985	(0.961–1.010)
Sex	0.892	0.956	(0.497–1.836)

ASA, American Society of Anesthesiologists; ESR, erythrocyte sedimentation rate; RDW-SD, red cell volume distribution width- standard deviation; NEUT, neutrophilic granulocyte count; ALB, albumin; AFR, albumin-fibrinogen ratio; CRP, C-reactive protein.

### Restrictive cubic spline regression analyses between preoperative albumin levels and acute postoperative infection

**Figure 2:**
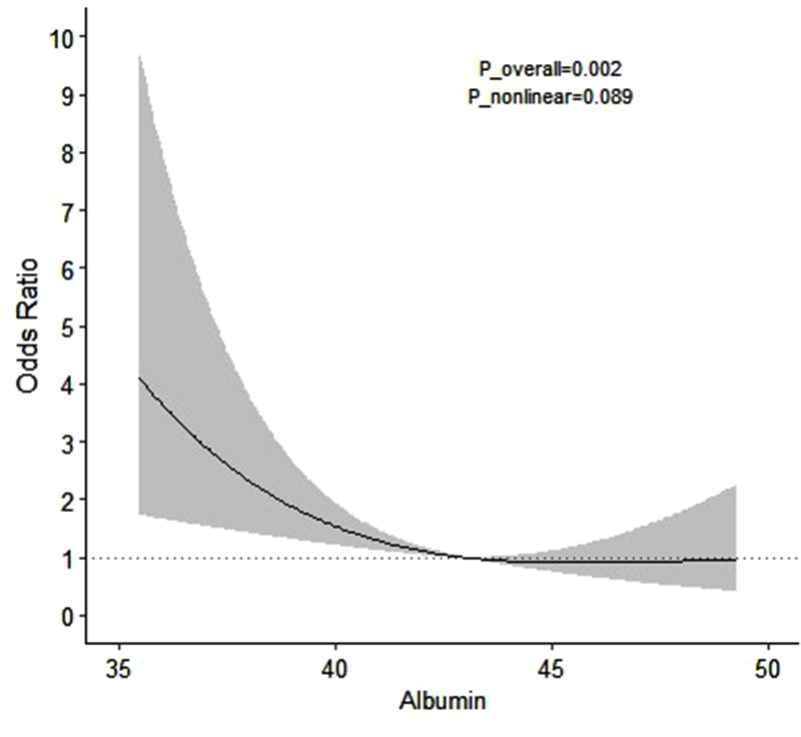
Dose-response relationship between preoperative albumin levels and the risk of acute postoperative infection.

We examined the dose-response relationship between preoperative serum albumin levels and acute postoperative infection by using restrictive cubic spline regression analysis. We found that the risk of acute postoperative infection increased with a decrease in preoperative albumin levels in a negative dose-response relationship (*P*_overall_ = 0.002; *P*_nonlinear_ = 0.089) (Figure 2).

### TKA and THA sub-analysis

The univariable analysis showed that serum albumin levels (*P* = 0.002), C-reactive protein concentrations (*P* = 0.042), Red Cell volume Distribution Width- standard deviation (*P* = 0.01), monocyte count (*P* = 0.037), smoking (*P* = 0.009) were associated with acute infection after primary TKA (Table 3). Multivariate logistic regression analysis showed that albumin levels (OR 0.880; 95% CI 0.776–0.998; *P* = 0.046) and smoking (OR 20.122; 95% CI 1.598–253.334; *P* = 0.020) were associated with acute infection after primary TKA (Table 4).

**Table 3:** Univariable analysis of risk factors for acute postoperative infection after TKA and THA.

Perioperative factors	*P* value (TKA)	*P* value (THA)
BMI (kg/m^2^)		
Underweight	1.00 (reference)	1.00 (reference)
Normal	1.00 (reference)	1.00 (reference)
Overweight	1.00 (reference)	1.00 (reference)
Obesity	0.293	0.386
ASA class		
I and II	1.00 (reference)	1.00 (reference)
III and IV	0.163	0.240
Hypertension		
No	1.00 (reference)	1.00 (reference)
Yes	0.459	0.871
Type 2 diabetes		
No	1.00 (reference)	1.00 (reference)
Yes	0.869	0.684
Smoking		
No	1.00 (reference)	1.00 (reference)
Yes	0.009*	0.113
Alcohol abuse		
No	1.00 (reference)	1.00 (reference)
Yes	0.715	0.517
Autoimmune disease		
No	1.00 (reference)	1.00 (reference)
Yes	0.162	0.267
Preoperative laboratories		
ESR (mm/h)	0.222	0.181
Hb (g/L)	0.116	0.496
RBC (10^12^/L)	0.127	0.717
RDW-SD (fl)	0.010*	0.838
RDW-CV (fl)	0.071	0.079
PLT (10^9^/L)	0.579	0.505
WBC (10^9^/L)	0.106	0.780
NEUT# (10^9^/L)	0.115	0.377
LYMPH (10^9^/L)	0.467	0.781
MONO (10^9^/L)	0.037*	0.838
ALB (g/L)	0.002*	0.012*
Globulin (g/L)	0.668	0.411
Fibrinogen (g/L)	0.297	0.482
AFR	0.054	0.711
CRP (mg/L)	0.042*	0.117

ASA, American Society of Anesthesiologists; BMI, body mass index; ESR, erythrocyte sedimentation rate; Hb, hemoglobin; RBC, red blood cell count; RDW-SD, red cell volume distribution width- standard deviation; RDW-CV, red cell volume distribution width-coefficient of variation; PLT, platelet count; WBC, white blood cell count; NEUT, neutrophilic granulocyte count; LYMPH, lymphocytes count; MONO, monocyte count; ALB, albumin; AFR, albumin-fibrinogen ratio; CRP, C-reactive protein.

* *P* < 0.05.

**Table 4:** Multivariable analysis of independent risk factors for acute postoperative infection after TKA and THA.

Perioperative factors	TKA	THA
	*P* value	OR	95% CI	*P* value	OR	95% CI
Age	0.741	0.994	(0.959–1.031)	0.508	0.988	(0.952–1.024)
Sex	0.534	1.409	(0.478–4.152)	0.974	0.985	(0.413–2.349)
ALB	0.046*	0.880	(0.776–0.998)	0.051	0.898	(0.806–1.000)
RDW-CV	0.756	0.935	(0.610–1.432)	0.059	0.716	(0.506–1.013)
RDW-SD	0.172	1.098	(0.960–1.254)	-		
AFR	0.934	1.006	(0.881–1.148)	-		
CRP	0.326	1.034	(0.967–1.105)	-		
MONO (10^9^/L)	0.561	2.390	(0.126–45.167)	-		
Smoking				-		
NO	1.00 (reference)	-	-	-		
YES	0.020*	20.122	(1.598–253.334)	-		

ALB, albumin; RDW-SD, red cell volume distribution width- standard deviation; RDW-CV, red cell volume distribution width-coefficient of variation; AFR, albumin-fibrinogen ratio; CRP, C-reactive protein; MONO, monocyte count.

* *P* < 0.05.

The univariable analysis showed that only serum albumin levels (*P* = 0.012) were associated with acute infection after primary THA. No significant associations were found between these indicators and acute infection after primary THA in multivariate analysis.

### SSI sub-analysis

The univariable analysis showed that serum albumin levels (*P* = 0.004) and C-reactive protein concentrations (*P* = 0.036) were associated with acute SSI (Table 5). The multivariate logistic regression analysis showed that only albumin levels (OR 0.897; 95% CI 0.820–0.981; *P* = 0.017) were associated with acute SSI (Table 6).

**Table 5:** Univariable analysis of risk factors for acute SSI after TJA.

Perioperative factors	Acute SSI (*n* = 63)	Non-infection (*n* = 126)	*P* value
BMI (kg/m^2^)			
Underweight	3 (4.8%)	6 (4.8%)	1.00 (reference)
Normal	18 (28.6%)	43 (34.1%)	1.00 (reference)
Overweight	10 (19.0%)	26 (20.6%)	1.00 (reference)
Obesity	30 (47.6%)	51 (40.5%)	0.810
ASA class			
I and II	58 (92.1%)	118 (93.7%)	1.00 (reference)
III and IV	5 (7.9%)	8 (6.3%)	0.684
Hypertension			
No	43 (68.3%)	84 (66.7%)	1.00 (reference)
Yes	20 (31.7%)	42 (33.3%)	0.827
Type 2 diabetes			
No	56 (88.9%)	113 (89.7%)	1.00 (reference)
Yes	7 (11.1%)	13 (10.3%)	0.867
Smoking			
No	60 (95.2%)	115 (91.3%)	1.00 (reference)
Yes	3 (4.8%)	11 (8.7%)	0.492
Alcohol abuse			
No	57 (90.5%)	108 (85.7%)	1.00 (reference)
Yes	6 (9.5%)	18 (14.3%)	0.354
Autoimmune disease			
No	53 (84.1%)	114 (90.5%)	1.00 (reference)
Yes	10 (15.9%)	12 (9.5%)	0.199
Preoperative laboratories			
ESR (mm/h)	32.84 ± 25.17	30.39 ± 19.65	0.464
Hb (g/L)	128.89 ± 22.91	135.63 ± 41.13	0.228
RBC (10^12^/L)	4.36 ± 0.49	4.44 ± 0.60	0.377
RDW-SD (fl)	46.84 ± 4.57	45.72 ± 5.10	0.144
RDW-CV (fl)	13.91 ± 1.82	13.98 ± 1.26	0.779
PLT (10^9^/L)	187.32 ± 80.93	191.67 ± 68.73	0.7
WBC (10^9^/L)	6.13 ± 1.85	5.97 ± 1.80	0.555
NEUT# (10^9^/L)	3.97 ± 1.67	3.70 ± 1.36	0.236
LYMPH (10^9^/L)	1.62 ± 0.60	1.66 ± 0.60	0.688
MONO (10^9^/L)	0.36 ± 0.14	0.34 ± 0.13	0.350
ALB (g/L)	41.56 ± 4.41	43.37 ± 3.50	0.004*
Globulin (g/L)	27.70 ± 5.38	28.82 ± 18.23	0.634
Fibrinogen (g/L)	3.13 ± 0.79	3.06 ± 0.74	0.548
AFR	14.24 ± 4.22	15.01 ± 3.85	0.224
CRP (mg/L)	9.22 ± 16.38	4.62 ± 6.87	0.036*

ASA, American Society of Anesthesiologists; BMI, body mass index; ESR, erythrocyte sedimentation rate; Hb, hemoglobin; RBC, red blood cell count; RDW-SD, red cell volume distribution width- standard deviation; RDW-CV, red cell volume distribution width-coefficient of variation; PLT, platelet count; WBC, white blood cell count; NEUT, neutrophilic granulocyte count; LYMPH, lymphocytes count; MONO, monocyte count; ALB, albumin; AFR, albumin-fibrinogen ratio; CRP, C-reactive protein.

* *P* < 0.05.

**Table 6:** Multivariable analysis of independent risk factors for acute SSI after TJA.

Perioperative factors	*P* value	OR	95% CI
ALB	0.017*	0.897	(0.820–0.981)
CRP	0.141	1.029	(0.991–1.069)
Age	0.297	0.986	(0.960–1.013)
Sex	0.883	1.060	(0.487–2.309)

ALB, albumin; CRP, C-reactive protein.

* *P* < 0.05.

No significant associations were found between these indicators and acute PJI after primary TJA in the univariable and multivariate analysis due to the overly small sample size of acute PJI (18 cases). Therefore, large sample studies are needed to analyze the risk factors of acute PJI.

## Discussion

Our study aimed to investigate various potential preoperative risk factors for acute infection after primary TJA. We especially focused on the patient's preoperative nutritional status such as albumin level, and AFR. We found that although there is currently insufficient evidence to confirm the relationship between preoperative AFR and acute infection after elective primary TJA, low albumin levels were still an independent risk factor for acute infection after TJA. Additionally, this risk increased with a decrease in preoperative albumin levels in a negative dose-response relationship. Most primary TJA is elective surgery. Therefore, our results emphasize the need for optimal nutritional management before surgery to reduce the risk of infection and unnecessary medical costs.

Acute infection after joint arthroplasty (e.g., PJI and SSI), which is defined as infection within 90 days after surgery, is a common complication of joint surgery (6, 18). PJI is one of the worst types of complications in hip and knee arthroplasty (5) and poses a large challenge to the medical community. Most patients with PJI require surgical intervention, usually multiple surgical procedures, which cause great physical pain to patients. PJI also causes an enormous financial burden to patients and resource use of hospitals and surgeons (19). SSI dramatically increases the risk of PJI, especially during the acute phase after surgery, because the articular cavity is exposed to a considerable number of infectious microorganisms (5).

Malnutrition is described as a situation in which an energy, protein, or other nutritional shortage or imbalance has observable negative consequences on body tissue, altered metabolism, and an increased risk of illness (20). In a laboratory test, malnutrition is defined as follows: albumin concentrations <3.5 mg/dl, total lymphocyte count <1,500/mm^3^, or serum transferrin concentrations <200 mg/dl (11, 21, 22). According to numerous studies, malnutrition is prevalent in older patients receiving TJA. Malnutrition is also associated with a considerable increase in postoperative complications, such as hematoma formation, and renal and cardiac complications (8, 9, 22–24). Albumin is an intuitive and sensitive marker, and it is most commonly used for nutritional screening (7, 22, 25). More importantly, albumin can be easily detected and corrected before elective surgery.

In addition to the serum albumin level being a reliable predictor to assess postoperative complications, it is also a risk factor for postoperative infection in orthopedic surgery. Kishawi et al. found substantial differences in 30-day postoperative complications after primary or revision TJA between patients with normal preoperative albumin concentrations and those with low albumin concentrations (7). Additionally, the rates of superficial and deep incisional surgical site infections were considerably increased in patients with low albumin concentrations. Daniel et al. also suggested that hypoalbuminemia was associated with an increased risk of SSI within 30 days following primary TJA (8). Their subsequent study showed that hypoalbuminemia was also associated with an increased risk of PJI in 30 days after revision TJA (24). Yi et al. reported that malnutrition was associated with an increased infection rate within 90 days after revision hip and knee surgery (9). Our study also showed that low albumin levels were an independent risk factor for acute infection (<90 days) after primary TJA. Albumin is synthesized by the liver. Nutrition and inflammation affect albumin synthesis (26). When patients suffer from malnutrition, their energy, protein, or amino acid supply is reduced, their albumin synthesis rate slows down, and serum albumin concentrations decrease (26, 27). Albumin also plays a crucial role in wound healing and immune function (28, 29). Therefore, the association between preoperative low albumin levels and acute postoperative infection is likely to be complex. One possible reason for this complex association is that patients with low albumin levels may suffer from a poor nutritional status and lack other vital vitamins and nutrients. This in turn could affect wound healing by decreasing collagen synthesis and fibroblast proliferation (7, 8, 28–30). Another possible explanation is that hypoalbuminemia might decrease inflammatory responses that would ordinarily combat infection (7, 9).

Fibrinogen, which is an acute-phase protein synthesized by the liver, rapidly increases in acute-phase conditions, such as bacterial infections and trauma (31). As a combined marker, the AFR is regarded as a predictive predictor for a variety of diseases. Recently, many studies have shown that the AFR has a great predictive value for the nutritional status and prognosis of disease. Claps et al. showed that a preoperative low AFR was a prognostic biomarker for worse time-to-progression, overall survival, and cancer-specific survival of patients with bladder cancer (32). Li et al. showed that the AFR can be used as a potential biomarker for predicting the patient's prognosis after renal cancer surgery, and improving the predictability of recurrence and survival (14). Zulipikaer et al. found that the AFR effectively reflected the nutritional, inflammatory, and coagulation status of patients undergoing revision TJA, and predicted acute PJI after revision TJA (16). Conversely, our study showed that there was no significant difference between the AFR and acute infection after primary TJA. In theory, using the AFR as a predictive marker may reduce the number of confounding factors that might arise when using albumin or fibrinogen alone. However, compared with the positive results of AFR in revision surgery by Zulipikaer et al. we believe that the physical and nutritional status of patients undergoing elective primary arthroplasty is much better than that of patients awaiting revision surgery. In the data of Zulipikaer et al. the proportion of patients with abnormally high fibrinogen levels (>4 g/L) was 25.1% in the aseptic revision cohort and as high as 68.3% in the septic revision cohort. In our two groups of patients, there was a small proportion (12.3% in the acute infection group and 8.6% in the uninfected group) of patients with abnormally increased levels of fibrinogen, and the abnormal values were only slightly higher than the normal high line value, so this will greatly affect the calculation of the combined index (AFR). Therefore, we think the conclusion that “AFR may be an effective biomarker to predict acute PJIs after revision TJA” could not be applied in elective primary joint arthroplasty. Indeed, our sample size was not large enough, which may result in a non-significant difference. Thus further studies with a larger sample size are required to confirm the relationship between the AFR and acute infection after primary TJA.

As for the univariate analysis, we found that patients with high preoperative CRP or who suffered from autoimmune diseases were associated with acute infection after primary TJA. The increased CRP before surgery indicates that the patient may be in an inflammatory state or have an occult infection somewhere in the body. Patients with autoimmune diseases (rheumatoid arthritis, ankylosing spondylitis) have long-term use of immune-suppressing drugs. Their immunity is often lower than normal, resulting in a higher risk of infection. However, these two potential risk factors were not statistically significant in the multivariate analysis. We believe this may be also related to the small sample size of this experiment.

Multivariate logistic regression analysis showed that albumin levels and smoking were associated with acute infection after primary TKA. Although our results showed smoking is significantly associated with acute infection after TKA, we still consider this needs to be proved with larger samples. Because females comprised the majority (76%) in our TKA cohort, and they have an extremely low smoking prevalence in contrast to the higher smoking rate among males in the TKA cohort. No significant associations were found between these indicators and acute infection after primary THA in multivariate analysis. But we still found that ALB may be a potential marker predicting acute infection after primary THA though did not reach a level of statistical significance (*P* = 0.051). In addition, the statistical trend was consistent with our previous results that albumin levels were associated with acute postoperative infection (OR 0.897; 95% CI 0.822–0.980; *P* = 0.015).

The multivariate logistic regression analysis showed that only albumin levels were associated with acute SSI. This result is similar to our previous results that combined PJI and SSI as acute postoperative infection. No significant associations were found between these indicators and acute PJI after primary TJA in the univariable and multivariate analysis due to the overly small sample size of acute PJI (18 cases). Therefore, large sample studies are needed to analyze the risk factors of acute PJI.

There are several limitations to this study. First, this study was retrospective and had the same biases inherent in other retrospective studies. Second, because of the low prevalence of acute PJI, there were only 18 acute PJI cases in this study. Third, owing to the limited overall sample size, the results of TKA and THA sub-analysis need to be further confirmed and must be interpreted with caution. Additionally, a well-designed, prospective study is required to further evaluate the predictive effect of the AFR on acute infection after primary arthroplasty.

## Conclusion

There is currently insufficient evidence to confirm the relationship between preoperative AFR and acute infection after elective primary TJA, while a lower preoperative albumin level is an independent risk factor for acute infection with a negative dose-response relationship. Therefore, optimal nutritional management may be necessary before elective primary TJA.

## Data Availability

The original contributions presented in the study are included in the article/Supplementary Material, further inquiries can be directed to the corresponding author.
